# Effect of Grape Pomace Flour in Savory Crackers: Technological, Nutritional and Sensory Properties

**DOI:** 10.3390/foods12071392

**Published:** 2023-03-24

**Authors:** Joana Marcos, Raquel Carriço, Maria João Sousa, M. Lídia Palma, Paula Pereira, M. Cristiana Nunes, Marisa Nicolai

**Affiliations:** 1 ECTS—Universidade Lusófona, Campo Grande 376, 1749-024 Lisboa, Portugal; 2 CBIOS—Research Center for Biosciences & Health Technologies, Universidade Lusófona, Campo Grande 376, 1749-024 Lisboa, Portugal; 3 CERENA—Center for Natural Resources and Environment, Instituto Superior Técnico (IST), Universidade de Lisboa, Av. Rovisco Pais, 1049-001 Lisboa, Portugal; 4 EPCV—Universidade Lusófona de Humanidades e Tecnologias, Campo Grande 376, 1749-024 Lisboa, Portugal; 5 LEAF—Linking Landscape, Environment, Agriculture and Food Research Center, Associated Laboratory TERRA, School of Agriculture, University of Lisbon, Tapada da Ajuda, 1349-017 Lisbon, Portugal

**Keywords:** wine industry, by-products, grape pomace, crackers, dietary fiber

## Abstract

The wine industry generates large amounts of by-products that are usually destined as waste. Grape pomace is the residue of the winemaking process and is rich in compounds with functional properties, such as dietary fiber and phenolic compounds. The aim of this research was to study the influence of white and red grape pomace flour (GPF) addition in the enhancement of functional properties of savory crackers. Different levels of incorporation were tested (5%, 10% and 15% (*w*/*w*)). Analysis of physical properties, nutritional composition and sensory acceptability were conducted to evaluate the effect of GPF incorporation. GPF cracker stability throughout a four-week period was achieved with regard to firmness and color. These products presented distinctive and appealing colors, ranging from a violet (GPF of Touriga Nacional variety) to a brown hue (GPF of Arinto variety). Concerning nutritional composition, both crackers incorporated with 10% GPF of Arinto or Touriga Nacional varieties could be considered “high in fiber”, as per the Regulation (EC) No. 1924/2006, suggesting a functional food. GPF crackers demonstrated an overall great acceptance of this kind of innovative foods, with the majority indicating that they would certainly/probably buy them. Moreover, the cracker with 10% Arinto GPF achieved the most balanced and overall preference.

## 1. Introduction

The food industry presents increasingly diversified offerings adapted to consumers’ demands. Moreover, consumers are increasingly more aware of the need to change their lifestyle behavior, aligned with a circular bioeconomy concept [1,2,3]. There has been exponential growth in the demand for foodstuffs with health-promoting ingredients, as well as the valorization of snacks, which are seen as practical and easy-to-consume products [4,5]. Furthermore, the application of practices that promote circular bioeconomy guidelines constitutes a strong determinant of individuals’ food choices, i.e., that allow the needs of current generations to be guaranteed without compromising the needs of future generations [6,7]. Therefore, the valorization of by-products is considered necessary and opportune to promote the development of new foods with added nutritional and economic value, in a reality of scarce resources [8,9,10].

Grapes are one of the most valued crops in the world, being mainly used for wine production [11,12]. Viticulture is one of the oldest economic sectors and represents one of the most important agricultural activities worldwide [13]. According to the International Organization of Vine and Wine (OIV), the total global production of wine in 2022 was estimated at around 260 million hectoliters [14]. Additionally, wine production is mainly located in Mediterranean countries, namely Italy, France, Spain, and Portugal [14]. Nevertheless, the technological processing of vinified grapes tends to result in different by-products, commonly labelled as waste [15,16,17,18]. However, these by-products have been widely associated with health benefits, providing compounds with functional properties [17,19].

Grape pomace is the residue that results from the pressing process of fresh grapes and represents 30% of the total quantity of vinified grapes worldwide (approximately 20 million tons/year) [16,17]. It constitutes 20%–25% of grape weight and is composed of skins, stems and seeds [15,20]. Traditionally, this by-product is used to produce fertilizer, feed, or distillates [16,21,22]. However, grape pomace has recently been seen as a promising alternative to obtain high value-added products [12,18,21,22]. Depending on the technology used in wine processing, two types of materials can be distinguished [20]: the fresh or sweet pomace not fermented from white wine contains a low alcoholic content and high levels of sugar; the red or fermented grape pomace contains a high alcoholic content and is obtained from the fermentation of solid residues in contact with the liquid portion [23,24]. Nevertheless, the intrinsic physicochemical characteristics of grape pomace are responsible for defining the quality of the wine and, consequently, the particularities of the residues obtained after the pressing process [20].

This by-product represents a complex substrate and is rich in bioactive compounds, such as dietary fiber, unsaturated fatty acids, minerals, vitamins, and antioxidants, mainly in the form of phenolic compounds (phenolic acids, flavonoids and proanthocyanins) [22,25,26]. Multiple studies during the last decade have shown that consumption of high-fiber foods, a characteristic of grape pomace, can reduce the risk of non-communicable diseases [27,28,29]. Moreover, different incorporation levels of grape pomace (red and white) in baked products (bread, cookies, and muffins) have been shown to increase its nutritional properties, mainly dietary fiber, and phenolic compounds [16,30,31,32]. Thus, the incorporation of grape pomace flour (GPF) in products traditionally consumed by the population, such as crackers, presents a possible strategy in the valorization of this bio-residue with potential health benefits [24,26]. However, the incorporation of grape pomace in food formulations can be a great challenge from a technological point of view [4,33].

This research aimed to study the effect of white and red grape pomace flour addition on technological aspects, nutritional composition, and sensory properties of savory crackers in order to enhance their functional properties. Different levels of GPF incorporation were tested (5%, 10% and 15% (*w*/*w*)).

## 2. Materials and Methods

### 2.1. Grape Pomace Flours

Grape pomace samples (*Vitis vinifera* L.) of the Arinto and Touriga Nacional varieties, from white and red wine production, respectively, were collected in Carmim, (Reguengos de Monsaraz, Alentejo, Portugal) in the year 2018. After collection, the samples were dried with an air circulation mechanism (J.P. Selecta, Barcelona, Spain) for 24 h at 60 °C and crushed in a blade grinder (Moulinex, Alencon, France). Grape pomace flours (GPF), with particle size fractions ≤400 μm, were used for all experiments. The samples were packed in properly sealed propylene bags and stored at 25 °C ± 2 °C for 4 weeks in a light-free place until analysis [20]. Nutritional characterization of both GPF varieties is shown in Table 1, according to incorporation level in the savory crackers.

### 2.2. Cracker Production

Considering a standard formulation established in a previous work from Batista et al. [34], the crackers were prepared using two grape pomaces of the species Vitis vinifera L., extra-fine wheat flour T55 (Nacional, Santarém, Portugal), baking powder (Royal, PA, USA), salt (Vatel, Alverca, Portugal), white sugar (Sidul, Santa Iria da Azóia, Portugal), sunflower oil (Fula, Barreiro, Portugal) and water. Six different formulations were prepared with incorporation of GPF: three formulations from the Arinto variety (A_1_, A_2_ and A_3_) and three other formulations from the Touriga Nacional variety (TN_1_, TN_2_ and TN_3_) at incorporation levels of 5%, 10% and 15% (*w*/*w*), respectively. The details of the various preparations are shown in Table 2.

**Figure 1:**
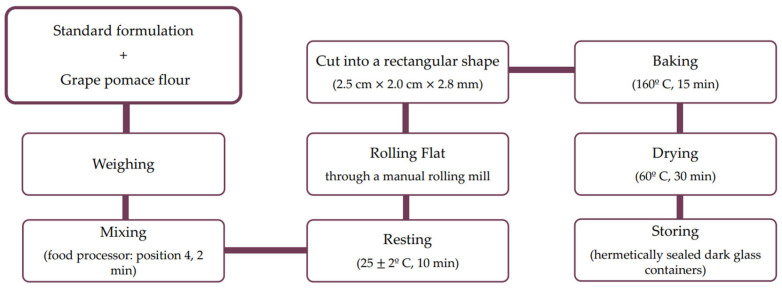
Flowchart for production of savory crackers with grape pomace flour.

The ingredients (total of 250 g) were weighed on a digital scale (Precisa BJ 1100D, Dietikon, Switzerland) and mixed in a food processor (Vorwek, Wuppertal, Germany) at position 4 for 2 min until a consistent and homogeneous dough was obtained. Subsequently, the dough was flattened in a manual rolling mill (Atlas Marcato, 150, Campodarsego, Italy) at positions 2, 4 and 6. This step was repeated three times for each position. Afterwards, the dough was cut into a rectangular shape (2.5 cm × 2.0 cm × 2.8 mm) and it was left to rise at 25 °C ± 2 °C for 10 min. The baking process took place in a forced air convection oven (Arianna Unox, Padua, Italy) at a temperature of 160 °C for 15 min. Finally, the crackers were dried in an electric oven (Arianna Unox, Padua, Italy) at a temperature of 60 °C for 30 min and then cooled at 25 °C ± 2 °C. After the confection process, the crackers were stored in dark glass containers, hermetically sealed, and properly identified. Each formulation was prepared in triplicate in approximate batches of 60 units. Production process can be seen in Figure 1.

### 2.3. Nutritional Analysis

Six different formulations, as well as a control sample, were developed. Once the preferred incorporation had been chosen (10% (*w*/*w*) for both grape pomace varieties (A_2_ and TN_2_), as shown in Palma et al. [35]), a nutritional evaluation was conducted. In this follow up, the preferred product was previously weighed, dried and stabilized at 50 °C [36].

The energy value was calculated in kcal from the proteins, lipids, fiber and carbohydrates content, using the follow equation [37]:

(1) $$Energy\;value\left(kcal\right)=4\times Proteins+9\times Lipids+4\times Carbohydrates+2\times Fiber$$

Protein content was determined by quantifying total nitrogen according to the methodology described in ISO 16634, using a conversion factor of 6.25 for crackers [38]. The determination of the lipid content was based on the pulsed nuclear magnetic resonance technique, by means of a calibration line. Total ash content was determined by incineration at 500 °C in a muffle furnace [39]. Total dietary fiber content was determined according to AOAC method 985.29 [40]. Carbohydrate quantification was performed indirectly, using the following equation [36]:

(2) $$Carbohydrates\left(\%\right)=100-(Proteins+Lipids+Fiber+Moisture+Ash)$$

Quantification of fatty acids (saturated, monounsaturated, and polyunsaturated) was conducted according to the EN ISO 12966-2, using GC-FID Shimadzu GC2010 (Shimadzu, Kyoto, Japan), Phenomenex ZEBRON ZB-WAX column (15 m × 0.25 mm × 0.25 µm) (Phenomenex, Tokyo, Japan) and fatty acid methyl ester mix C4-C24 (SigmaAldrich, Darmstadt, Germany) as standard. The fat content present in the cracker was converted into free fatty acids by saponification (microwave action), and later converted into methyl esters after treatment with chloride acid and methanol. Finally, these methyl esters were extracted and identified by gas chromatography [41].

### 2.4. Physical Properties

A texture profile analysis was performed using a texture analyzer TA-XTplus Texturometer (Stable Micro Systems, Godalming, UK), equipped with a diameter cylindrical probe (SMSP/2SP) with a 2 mm diameter, in a room at a controlled temperature (20 °C ± 2 °C). The measurement conditions were 2 mm of penetration at a speed of 1 mm/s. Texture measurement was performed in replicate, 10 times per formulation, at different times: t_0_ (day of preparation, 2 h after baking), t_1_ (1st week), t_2_ (2nd week), t_3_ (3rd week) and t_4_ (4th week). Texture evaluation was displayed in terms of firmness, the maximum force, with results expressed in Newton (N).

Colorimetric properties were measured on the surface of the crackers, using a colorimeter (Minolta CR-400, Osaka, Japan), under specific conditions: standard illumination source D65 and visual angle of 2°, previously calibrated (L* = 94.61; a* = −0.53; b* = 3.62). The measurements were performed in triplicate per formulation at 25 °C ± 1 °C, under constant artificial light, at different times. The results were expressed using the CIELab system, through the following parameters: L*—brightness (L* = 100 white; L* = 0 black), a*—intensity of green (−60 < a* < 0) or red (0 < a* < +60) and b*—intensity of blue (−60 < b* < 0) or yellow (0 < b* < +60). Saturation of color (C*) was calculated using the following equation: $C^{*}=\sqrt{\left(a^{2}+b^{2}\right)}$. The total difference of colorimetric properties (ΔE*) was evaluated through the equation: ${∆E}^{*}=\sqrt{{\left({\Delta L}^{*}\right)}^{2}+{\left({\Delta a}^{*}\right)}^{2}+{\left({\Delta b}^{*}\right)}^{2}}$. The hue angle value (h*) was calculated using the following equation: $h^{*}=\arctan\left(b^{*}/a^{*}\right)$.

The water activity (A_w_) was measured in triplicate for each formulation, 2 h after baking, with a thermohydrometer (HydroPalm HP23-Aw Rotronic, Bassersdorf, Switzerland), at a constant temperature of 25 °C ± 1 °C.

### 2.5. Sensory Evaluation

A sensory analysis was conducted under controlled laboratory conditions, according to the standard ISO 8589:2007 [42]. To determine the predilected typology of the grape pomace crackers, two formulations with 10% (*w*/*w*) grape pomace flour (A_2_ and TN_2_) and a control sample (X_0_) were evaluated, being presented simultaneously to the panel. A hedonic scale test (1 = *“very unpleasant”*, 2 = *“unpleasant”*, 3 = *“indifferent”*, 4 = *“pleasant”*, 5 = *“very pleasant”*) was conducted to evaluate sample acceptability, regarding color, flavor, texture, aroma, and global appreciation (five levels). Purchase intention was also assessed at five levels (1 = *“certainly would not buy”*, 2 = *“probably would not buy”*, 3 = *“indifferent”*, 4 = *“would probably buy”*, 5 = *“would certainly buy”*). Sensory analysis was evaluated through an untrained panel (*n* = 60; gender: females 31, males 29; age: 18–59 years (18–24 years: 48.3%, 25–29 years: 15.0%, 30–39 years: 13.3%, >39 years: 23.3%); 76.7% had higher education), under the terms established by the Declaration of Helsinki. 

### 2.6. Statistical Analysis

Data are expressed as means (±standard deviation (SD)) for continuous variables and absolute and relative frequencies (%) for categorical variables. Means of variables were compared by independent-sample Student’s *t*-test. One-way ANOVA was used to compare the means of more than two groups, followed by Tukey’s honestly significant difference (HSD) post hoc test. Statistical analysis was performed using the SPSS statistical package version 28.0 (IMB Inc., Armonk, NY, USA), with results considered statistically significant when *p* < 0.05. 

## 3. Results and Discussion

### 3.1. Physical Properties

#### 3.1.1. Texture Profile

**Figure 2:**
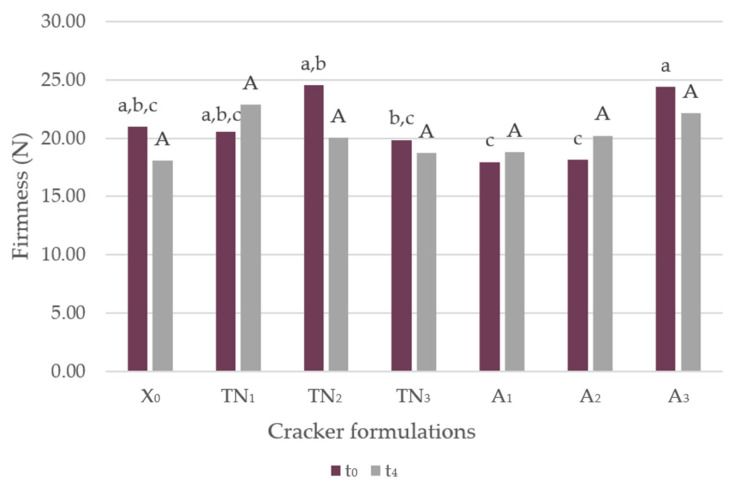
Firmness (N) of the savory crackers at t_0_ and t_4_. Different letters correspond to significant differences (ANOVA test Tukey, *p* < 0.05). Abbreviations: X_0_, control sample; TN_1_, Touriga Nacional with 5% incorporation; TN_2_, Touriga Nacional with 10% incorporation; TN_3_, Touriga Nacional with 15% incorporation; A_1_, Arinto with 5% incorporation; A_2_, Arinto with 10% incorporation; A_3_, Arinto with 15% incorporation; t_0_, day of confection; t_4_, 4th week after confection. The lower cases letters correspond to t_0_ and the capital letters correspond to t_4_.

The impacts of adding grape pomace flour (GPF), at various degrees of incorporation, at the time of confection (t_0_) and four weeks later (t_4_) are presented in Figure 2.

Traditionally, wheat flour is the main ingredient in most cookie formulations. According to the literature, this type of flour contains a high starch content (70%–75%) and about 8%–11% protein, all of which contribute strongly to the texture, more specifically hardness and shape of these food products [43]. The hydration of wheat flour with a combined mechanical energy forms the three-dimensional gluten network that confers great viscoelastic attributes to doughs, resulting in cookies with high firmness [44]. The baking properties can be significantly affected when developing products where part of the wheat flour is replaced by other ingredients [45]. Therefore, replacing part of the wheat flour with GPF will result in a decrease in gluten formation and starch content, but an increase in fiber. This proved to be accurate in terms of consumers’ acceptance, given that, in a previous sensory analysis, the texture of the GPF-incorporated crackers (5%, 10% and 15%) was preferred over the control [35]. Fiber is a crucial component of crackers, influencing their texture. Higher fiber content tends to inhibit dough hydration, as well as disrupt the gluten network formation, resulting in crackers with higher firmness levels [46]. According to Table 1, the Arinto variety GPF had a lower content of fiber, compared to the Touriga Nacional variety. Thus, the Arinto samples presented a lower value of firmness than the Touriga Nacional variety. No statistically significant differences (*p* > 0.05) were observed between the various degrees of incorporation of GPF and the control sample (without GPF, designated as X_0_), both at t_0_ and t_4_, with regard to firmness.

Concerning the texture parameters of the savory crackers over time, firmness was evaluated in each sample for a period of four weeks, as shown in Table 3.

Nonetheless, it was possible to note a trend associated with a significant increase in firmness with a 15% incorporation level at t_0_, compared to 5% and 10%. Furthermore, all samples displayed a firmness stability over time.

Numerous researchers have observed that when wheat flour is replaced by another flour with high fiber content, the dough after baking tends to increase in firmness (as previously stated) and volume, as well as show improved oxidative stability and prolonged shelf life, during the storage period [45,47,48,49,50]. An incorporation up to 15% of both the Arinto and Touriga Nacional GPF varieties showed no firmness disparities in the crackers. Moreover, storage in a closed package, which is equivalent to an at home storage environment, for a period of four weeks did not lead to considerable changes in firmness. 

#### 3.1.2. Color Profiling

**Figure 3:**
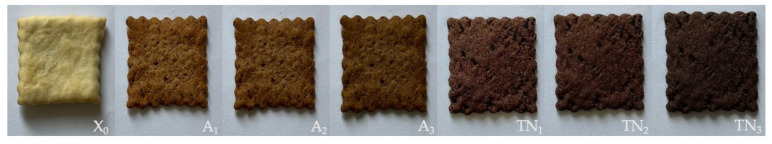
Control and 5%, 10% and 15% GPF-incorporated crackers from two different grape varieties. Abbreviations: X_0_, control sample; TN_1_, Touriga Nacional with 5% incorporation; TN_2_, Touriga Nacional with 10% incorporation; TN_3_, Touriga Nacional with 15% incorporation; A_1_, Arinto with 5% incorporation; A_2_, Arinto with 10% incorporation; A_3_, Arinto with 15% incorporation.

The GPF crackers presented visually attractive colors, ranging from a violet hue in the Touriga Nacional GPF crackers to brown in those of the Arinto variety, as shown in Figure 3. 

**Figure 4:**
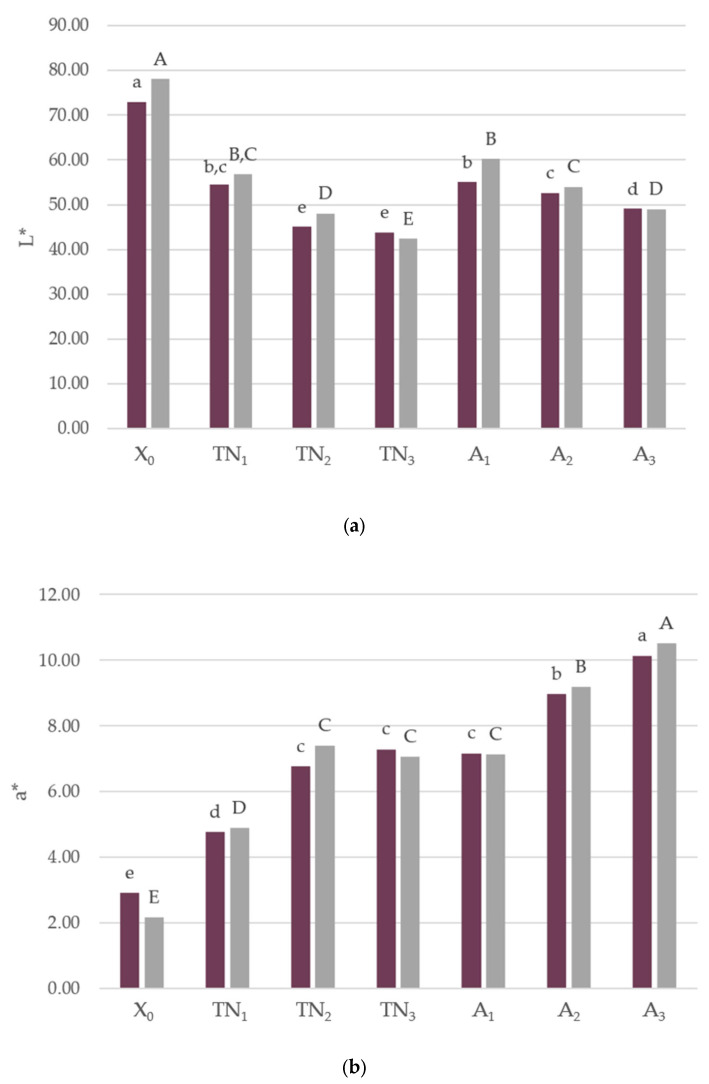

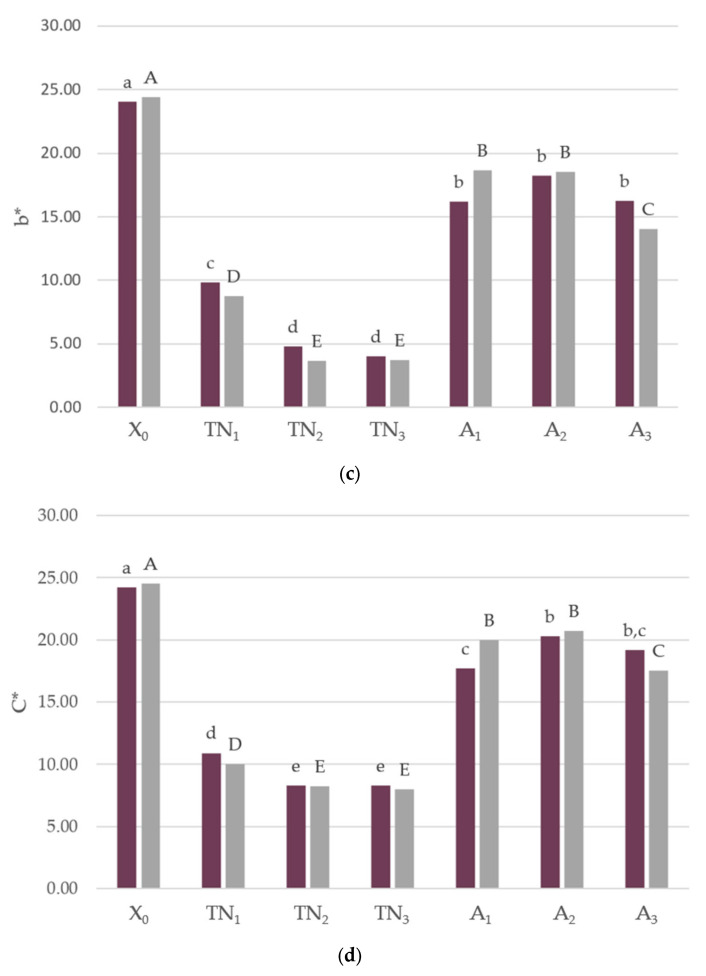

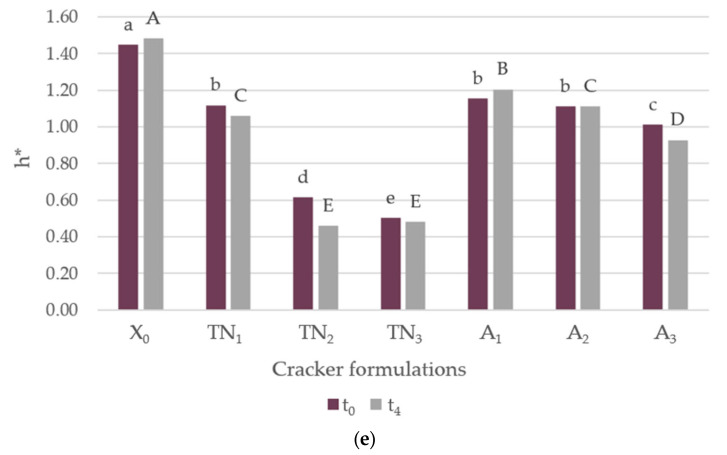
Color parameters L* (**a**), a* (**b**), b* (**c**), C* (**d**) and h* (**e**) of the savory crackers, at t_0_ and t_4_. Different letters correspond to significant differences (ANOVA test Tukey, *p* < 0.05). Abbreviations: X_0_, control sample; TN_1_, Touriga Nacional with 5% incorporation; TN_2_, Touriga Nacional with 10% incorporation; TN_3_, Touriga Nacional with 15% incorporation; A_1_, Arinto with 5% incorporation; A_2_, Arinto with 10% incorporation; A_3_, Arinto with 15% incorporation; t_0_, baking day; t_4_, 4th week after baking; L*, lightness; a*, yellowness; b*, greenness; C*, chroma; h*, hue. The lower cases letters correspond to t_0_ and the capital letters correspond to t_4_.

Figure 4 illustrates the results of the color measurement of the savory crackers with different levels of incorporation of grape pomace flour (GPF) of both the Touriga Nacional and Arinto varieties, at both the time of confection and on the fourth week of storage.

In terms of brightness of the crackers, it was observed that the L* values reduced significantly (*p* < 0.05) with increased GPF content, at both t_0_ and t_4_. As expected, the L* for the control vs. the other formulations was significantly reduced. Regarding a*, a predominance of red over green intensity was noted in all samples, as demonstrated by a positive a*. Furthermore, a significantly direct association was noted between a* values and GPF incorporation, at both t_0_ and t_4_. Moreover, it was observed that the addition of GPF presented significantly higher a* values than the control sample. Concerning b* values, all samples displayed a positive b*, indicating a higher yellow intensity in X_0_ (control) and samples of the Arinto GPF variety, however the increased incorporation of different levels of this variety did not appear to impact b* values. Similar behaviors were observed for the hue angle, with b* for the Touriga GPF variety and h* values decreasing significantly with the increased substitution of part of the wheat flour with GPF, both at t_0_ and t_4_. Interestingly, a parallel saturation was achieved between samples X_0_ and A_2_. A 10% incorporation level of GPF had a significant impact on C* when compared to 5%, however a stability of saturation was achieved at 10% with no visible differences for 15%.

Overall, as far as color coordinates are concerned, it can be stated that a reduction in L* and h* values was observed. Pertaining to saturation, color stability was displayed upon a 10% incorporation level. These results are supported by studies that evaluated colorimetric parameters in cookies with grape flour incorporated at levels between 5% [51] and 40% [52].

Color differences in visual perception of two given colors refers to ΔE* [53,54]. Thus, Table 4 presents the results of total color differences between control savory crackers and incorporated GPF of both varieties, as well as comparison amongst the previous level of incorporation.

Numerous researchers have indicated that the human eye is only able to perceive variations in color when ∆E* > 5 [55]. All GPF crackers, except A1 (∆E* = 5.8), displayed color stability throughout the four-week storage period, with variations being barely detected by the human eye. On the other hand, the control sample revealed a ∆E* of 5.2 which suggests a color change during the storage period. What is more, the incorporation of 10% or 15% of the Touriga Nacional GPF (∆E* = 1.6) revealed no apparent impact on color at the moment of confection. Likewise, the increased incorporation of the Arinto GPF variety (5%, 10% and 15%) did not seem to present an impact on the color of the crackers.

#### 3.1.3. Water Activity and Moisture

To better study the relation between overall product quality parameters and raw materials, water activity (aw) and moisture of the savory crackers were evaluated (Table 5).

The incorporation of grape pomace flour (GPF) was related to increased water activity in the developed crackers, with significant increases (*p* < 0.05) only observed for samples TN_2_ (0.34) and A_1_ (0.31), when compared to the control (X_0_). As per scientific evidence, there should be no differences in the risk of microbial growth during storage, nor lipid oxidation and enzymatic changes [56,57].

Regarding the moisture values of the developed savory crackers, no statistically significant differences (*p* > 0.05) were observed between the different formulations and the control. However, an indirect relationship between the moisture values and the fiber content, associated with the incorporation of grape GPF was noted; in other words, there was a significant decrease in moisture from A_1_ (5.33%) to A_3_ (3.41%). Furthermore, a study on the functional value of grape pomace found reduced moisture content (1.89%–1.94%) when incorporating 2%, 4%, 6% and 8% GPF into cookies, which resulted in increased product stability [58], corroborating the results found here. 

Moreover, moisture loss and lower water activity upon baking can strongly influence the color profile and improve the stability of the crackers’ quality over time [46], as previously observed.

### 3.2. Nutritional Analysis

According to the article published in 2020 that used the same formulations [35], a clear preference was highlighted for the samples labelled as TN_2_ and A_2_, i.e., savory crackers with 10% incorporation of both Touriga Nacional and Arinto varieties, respectively. Thus, a nutritional analysis was carried out for the selected samples, as well as the control one (without GPF, X_0_), as shown in Table 6.

Regarding the samples with added GPF, these displayed a significantly higher content of fiber (9.40 g ± 2.40 g for TN_2_, 8.60 g ± 2.20 g for A_2_), in contrast to the control sample (1.60 g ± 1.20 g). Thus, and as per the Commission Regulation (EC) N.° 1924/2006 on nutrition and health claims made on foods [60], it can be claimed that both food products designated as TN_2_ and A_2_ are “high in fiber”, as opposed to X_0_. Moreover, studies have attributed the increased firmness of cookies with added GPF (10%–20% incorporation) to the increased fiber content from the composition of the pomace [4,29].

Grape pomace is described in literature as a predominant source of dietary fiber. Red grape pomaces tend to have higher fiber content, in comparison to white grape pomaces [21,61]. Thus, a lower fiber content was shown for A_2_ than for TN_2_. The largest nutritional component of grape pomace is reported as carbohydrates, as shown in Table 6. Several authors declared a lower soluble sugar content, mainly glucose and fructose, in red grape pomace, due to the red wine production process, which includes fermentation, where these sugars are largely used as primary energy sources fir yeasts [61]. On the other hand, the soluble sugar content found in white grape pomace tends to be higher, up to 26.34 g/100 g of glucose and 8.91 g/100 g of fructose, as it does not go through ethanolic fermentation. These increased sugar values appear to be responsible for the enhanced sweet taste in multiple products [22,29]. Surprisingly, the carbohydrate contents in A_2_ (66.04 g ± 0.56 g) and TN_2_ (67.02 g ± 0.62 g) were similar, however these do not refer simply to simple sugars, i.e., glucose and fructose. Nonetheless, the maximum sugar content varies depending on the grape variety but can be influenced by climatic factors. In 2018, wine production in Portugal was reported to be the lowest ever, with an output of 6.1 million hectoliters. This decrease in production was due to increased rainfall, influencing the sugar levels present in the grapes and, consequently, in the grape pomace [62,63].

### 3.3. Sensory Evaluation

**Figure 5:**
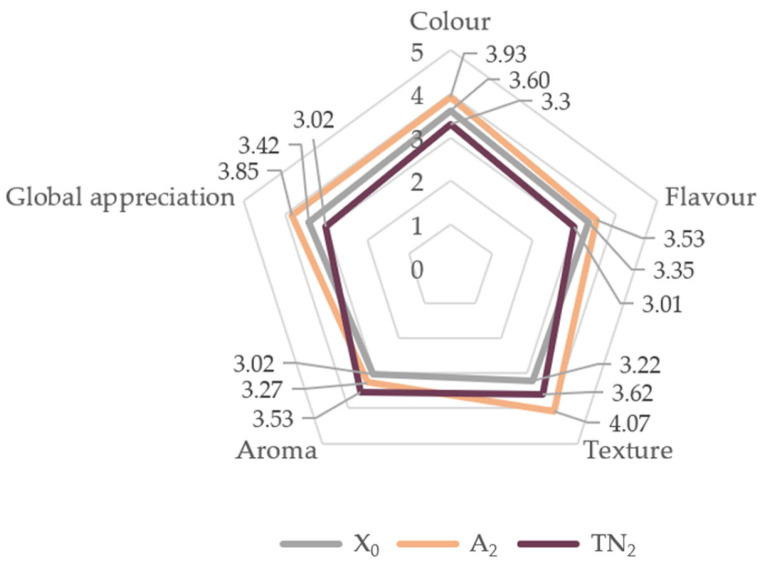
Sensory analysis (*n* = 60) of the savory crackers, concerning color, aroma, flavor, texture and global appreciation parameters. 1—*“*very unpleasant”; 2—“unpleasant”; 3—“indifferent”; 4—“pleasant”; 5—“very pleasant”. Abbreviations: X_0_, control sample; TN_2_, Touriga Nacional with 10% incorporation; A_2_, Arinto with 10% incorporation.

Sensory analysis assays were carried out for the crackers with grape pomace of the Touriga Nacional and Arinto varieties, at 10% incorporation level. Figure 5 indicates the average scores of the sensorially assessed parameters. 

All samples demonstrated a favorable acceptance (>3.0), indicating a good overall acceptability of GPF-incorporated crackers. A_2_ scored the highest values in terms of color, flavor, texture, and global appreciation, which can be associated with the instrumental texture and water activity results, suggesting a crunchy, aerated and more stable cracker. Furthermore, the overall acceptability of A_2_ with regard to color could be explained by a consumers’ preference for crackers with a yellow hue, instead of a violet one [58]. As regards flavor, the preference for A_2_ is not surprising due to the enhanced sweetness characteristic of the white grape variety, as opposed to the reported bitterness, astringency and aftertaste characteristics of products developed from red grape varieties, such as TN_2_. However, regarding the aroma, a preference for TN_2_ was noted, possibly due to the more intense, and sour aroma of the red grape varieties, in contrast to A_2_, as these grape varieties tend to be described as having a sweeter odor [64,65]. Moreover, differently to the results reported by Palma et al. (2020) [35], the TN_2_ samples showed lower appreciation on most sensory attributes, possibly due to the way the samples were presented to the consumer panel, i.e., simultaneously.

**Figure 6:**
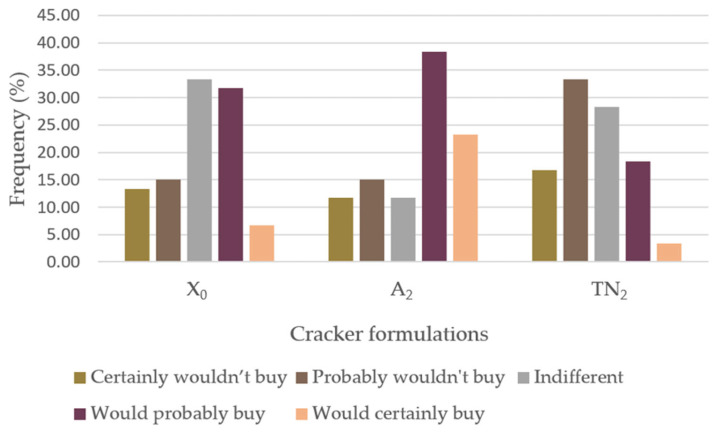
Sensory analysis (*n* = 60) of the savory crackers, concerning purchase intention. 1—“certainly would not buy”; 2—“probably would not buy”; 3—“indifferent”; 4—“would probably buy”; 5—“would certainly buy—”. Abbreviations: X_0_, control sample; TN_2_, Touriga Nacional with 10% incorporation; A_2_, Arinto with 10% incorporation.

Figure 6 represents the panelists’ buying intention. The majority (65%) of the participants stated that they were indifferent or would probably buy the X_0_ cracker formulation. An A_2_ preference was noted by the panel, given that 61.6% would certainly/probably buy. Regarding TN_2_, 61.6% of the individuals indicated that they were indifferent or that they probably would not buy. 

A_2_ crackers were reported as the crunchiest, tastiest and the most balanced regarding flavor, when compared to TN_2_ and X_0_. Hence, these attributes could explain a higher buying intention for the sample A_2_. Nonetheless, a good overall acceptance was verified for crackers with these innovative characteristics.

## 4. Conclusions

The incorporation of grape pomace flour from the Touriga Nacional and Arinto grape varieties in savory crackers does not seem to compromise their texture, in terms of firmness. This new food product presents innovative colors, ranging from violet in the crackers with Touriga Nacional to brown in those of the Arinto variety, which were perceptible to the human eye, when compared to the control ones. Thus, it was noted that during four weeks of storage at room temperature, in a closed package and protected from light, the crackers maintained stable texture and color. Moreover, the replacement of some of the wheat with GPF resulted in a decrease in gluten and starch content, but a significant increase in fiber, therefore it can be stated that these innovative crackers appear to have characteristic properties of a functional food. Furthermore, in the sensory evaluation, a preference was clearly noted for the Arinto variety cracker, when compared to Touriga Nacional variety and control crackers. Future studies should analyze the association between the consumption of food products incorporated with grape pomace flour and the possible impacts on human health.

## Figures and Tables

**Table 1:** Nutritional characterizations of grape pomace flour (GPF) per incorporation level in the crackers.

	TN_1_	TN_2_	TN_3_	A_1_	A_2_	A_3_
Protein, g	0.51	1.01	1.52	0.42	0.84	1.26
Lipids, g	0.41	0.81	1.22	0.56	1.11	1.67
Carbohydrates, g	0.21	0.43	0.64	1.33	2.66	3.99
Fiber, g	3.40	6.80	10.20	2.05	4.10	6.15
Ash, g	0.27	0.55	0.82	0.20	0.40	0.60
Moisture, g	0.20	0.40	0.60	0.89	1.77	2.66

Data expressed as mean. Abbreviations: TN_1_, Touriga Nacional with 5% incorporation; TN_2_, Touriga Nacional with 10% incorporation; TN_3_, Touriga Nacional with 15% incorporation; A_1_, Arinto with 5% incorporation; A_2_, Arinto with 10% incorporation; A_3_, Arinto with 15% incorporation.

**Table 2:** Detailed description of the crackers’ formulations (%, *w*/*w*), prepared from the incorporation of various percentages of Arinto and Touriga Nacional GPF.

	Ingredients, %							
Samples	Wheat Flour	GPF (Arinto)	GPF (Touriga Nacional)	Water	Baking Powder	Sunflower Oil	Salt	Sugar
**X_0_**	60.5	-	-	28.5	1.5	7.5	1	1
**TN_1_**	55.5	-	5	28.5	1.5	7.5	1	1
**TN_2_**	50.5	-	10	28.5	1.5	7.5	1	1
**TN_3_**	45.5	-	15	28.5	1.5	7.5	1	1
**A_1_**	55.5	5	-	28.5	1.5	7.5	1	1
**A_2_**	50.5	10	-	28.5	1.5	7.5	1	1
**A_3_**	45.5	15	-	28.5	1.5	7.5	1	1

Abbreviations: X_0_, control sample; TN_1_, Touriga Nacional with 5% incorporation; TN_2_, Touriga Nacional with 10% incorporation; TN_3_, Touriga Nacional with 15% incorporation; A_1_, Arinto with 5% incorporation; A_2_, Arinto with 10% incorporation; A_3_, Arinto with 15% incorporation.

**Table 3:** Firmness of the savory crackers throughout the four weeks of storage (t_0_, t_1_, t_2_, t_3_ and t_4_).

	t_0_	t_1_	t_2_	t_3_	t_4_
X_0_	20.96 ^a^ ± 4.75	19.26 ^a^ ± 3.46	21.85 ^a^ ± 3.63	20.01 ^a^ ± 4.49	18.08 ^a^ ± 5.41
TN_1_	17.96 ^c^ ± 3.90	21.45 ^b^ ± 2.31	23.19 ^a^ ± 8.00	18.47 ^a^ ± 3.32	18.80 ^b,c^ ± 4.39
TN_2_	18.15 ^c^ ± 2.99	22.42 ^b^ ± 3.53	20.35 ^b,c^ ± 4.57	27.98 ^a^ ± 2.99	20.21 ^b,c^ ± 3.40
TN_3_	24.39 ^b,c^ ± 4.82	29.18 ^a^ ± 4.70	16.96 ^b^ ± 3.37	23.54 ^b,c^ ± 3.69	22.13 ^c^ ± 5.59
A_1_	20.56 ^a^ ± 6.45	18.68 ^a^ ± 4.97	19.83 ^b,c^ ± 4.02	19.67 ^a^ ± 6.05	22.86 ^a^ ± 3.49
A_2_	24.52 ^a^ ± 4.74	17.54 ^c^ ± 3.55	20.89 ^b,c^ ± 4.51	22.06 ^a,b^ ± 3.96	20.05 ^b,c^ ± 4.01
A_3_	19.85 ^a,b^ ± 4.01	18.54 ^a,b^ ± 4.46	27.27 ^a,b^ ± 3.91	21.17 ^a^ ± 2.96	18.74 ^a,b^ ± 3.52

Data expressed as mean ± SD. Different letters for each row correspond to significant differences (ANOVA test Tukey, *p* < 0.05). Abbreviations: X_0_, control sample; TN_1_, Touriga Nacional with 5% incorporation; TN_2_, Touriga Nacional with 10% incorporation; TN_3_, Touriga Nacional with 15% incorporation; A_1_, Arinto with 5% incorporation; A_2_, Arinto with 10% incorporation; A_3_, Arinto with 15% incorporation; t_0_, day of confection; t_1_, first week after confection; t_2_, second week after confection; t_3_, third week after confection; t_4_, fourth week after confection.

**Table 4:** Total color difference of the savory crackers with grape pomace flour at different levels of incorporation, between t_0_ and t_4_, as well as comparison with the previous sample at t_0_.

	ΔE*	
	t_0_ vs. t_4_	Comparison with Previous Sample (t_0_)
X_0_	5.2	-
TN_1_	2.5	-
TN_2_	3.3	10.9
TN_3_	1.5	1.6
A_1_	5.8	-
A_2_	1.4	3.6
A_3_	2.3	4.2

Data expressed as mean. Abbreviations: ΔE*, total color difference; X_0_, control sample; TN_1_, Touriga Nacional with 5% incorporation; TN_2_, Touriga Nacional with 10% incorporation; TN_3_, Touriga Nacional with 15% incorporation; A_1_, Arinto with 5% incorporation; A_2_, Arinto with 10% incorporation; A_3_, Arinto with 15% incorporation; t_0_, day of confection; t_4_, fourth week after confection.

**Table 5:** Water activity (aw) and moisture values of savory crackers at the moment after confection (t_0_).

	Aw	Moisture (%)
X_0_	0.17 ^c^ ± 0.02	4.12 ^a,b^ ± 0.58
TN_1_	0.21 ^c^ ± 0.05	2.72 ^b^ ± 0.74
TN_2_	0.34 ^a^ ± 0.06	3.71 ^a,b^ ± 0.74
TN_3_	0.27 ^a,b,c^ ± 0.01	3.99 ^a,b^ ± 0.28
A_1_	0.31 ^a,b^ ± 0.00	5.33 ^a^ ± 0.99
A_2_	0.23 ^b,c^ ± 0.05	4.08 ^a,b^ ± 0.94
A_3_	0.25 ^a,c^ ± 0.01	3.41 ^b^ ± 0.19

Data expressed as mean ± SD. Different letters correspond to significant differences (ANOVA test Tukey, *p* < 0.05). Abbreviations: X_0_, control sample; TN_1_, Touriga Nacional with 5% incorporation; TN_2_, Touriga Nacional with 10% incorporation; TN_3_, Touriga Nacional with 15% incorporation; A_1_, Arinto with 5% incorporation; A_2_, Arinto with 10% incorporation; A_3_, Arinto with 15% incorporation; aw, water activity.

**Table 6:** Nutritional values associated with the formulation of savory crackers incorporating 10% grape pomace flour, compared to the control (X_0_).

	X_0_			TN_2_			A2		
	Per 100 g	Per Cracker	RDA (Per Cracker)	Per 100 g	Per Cracker	RDA (Per Cracker)	Per 100 g	Per Cracker	RDA (Per Cracker)
Energy, kcal	409.21	17.05	0.85	400.90	16.71	0.84	397.10	16.55	0.83
Protein, g	9.44 ± 0.35	0.39	0.79	8.57 ± 0.14	0.36	0.71	9.63 ± 0.07	0.40	0.80
Lipids, g	8.01 ± 0.64	0.33	0.48	8.86 ± 0.71	0.37	0.53	8.58 ± 0.69	0.36	0.51
of which saturated	0.82 ± 0.19	0.03	0.17	1.03 ± 0.23	0.04	0.21	0.99 ± 0.22	0.04	0.21
of which monounsaturated	2.72 ± 0.46	0.11	0.28	2.58 ± 0.44	0.11	0.27	2.48 ± 0.42	0.10	0.26
of which polyunsaturated	4.43 ± 0.71	0.18	0.92	5.25 ± 0.84	0.22	1.09	5.11 ± 0.82	0.21	1.06
Carbohydrates, g	74.04 ± 0.68	3.09	1.19	67.02 ± 0.62	2.79	1.07	66.04 ± 0.56	2.75	1.06
Fiber, g	1.60 ± 1.20	0.07	0.22	9.40 ± 2.40	0.39	1.31	8.60 ± 2.20	0.36	1.19
Ash, g	2.79 ± 0.10	-	-	3.58 ± 0.02	-	-	3.85 ± 0.09	-	-

Data expressed as mean ± SD. Abbreviations: X_0_, control sample; TN_2_, Touriga Nacional with 10% incorporation; A_2_, Arinto with 10% incorporation; RDA, recommended daily allowance for an average adult, i.e., 2000 kcal or 8400 kJ [59].

## Data Availability

Not applicable.
